# Head and neck squamous cell carcinoma with femoral metastasis: a case report and systematic review of current literature

**DOI:** 10.3389/fonc.2026.1807366

**Published:** 2026-07-06

**Authors:** Hannah Chahal, Joseph Latham, Finlay Ajayi, Sasan Dehbozorgi, Andrew Miller, Gordon Gillespie

**Affiliations:** Department of Trauma and Orthopaedics, The Grange University Hospital, Wales, United Kingdom

**Keywords:** bone metastases, femoral metastasis, head and neck (H&N) cancer, squamous cell carcinoma, tongue squamous cancer

## Abstract

Head and neck squamous cell carcinoma (HNSCC) predominantly spreads locoregionally, while distant metastases are uncommon and typically involve the lungs, liver, or axial skeleton; femoral metastasis is exceptionally rare. This study reports a case of femoral shaft metastasis arising from tongue squamous cell carcinoma and presents a systematic review of the existing literature to characterise clinical features, management, and outcomes. A systematic search of PubMed, Scopus, ScienceDirect, the British Medical Journal, and Google Scholar was conducted in accordance with PRISMA guidelines, identifying English-language case reports describing histologically confirmed HNSCC with femoral metastasis. Five published cases were identified and, together with the present case, six cases were analysed. The mean age at diagnosis was 57 years, with a male predominance (66.7%). The tongue was the most common primary tumour site (66.7%), and smoking was the most frequently reported risk factor. Time to femoral metastasis ranged from six months to 19 years, with most cases demonstrating early recurrence and advanced disease with nodal involvement or additional distant metastases. Management following femoral involvement was predominantly palliative and included radiotherapy, chemoradiotherapy, and orthopaedic stabilisation for impending or pathological fracture. Overall prognosis was poor, reflecting aggressive tumour biology and limited treatment options once distant skeletal disease is established. Femoral metastasis from HNSCC represents an advanced and uncommon disease manifestation, and early recognition is essential to facilitate timely palliative intervention, prevent skeletal complications, and optimise quality of life. Further multicentre studies and pooled analyses are required to better define risk factors, metastatic pathways, and optimal management strategies.

## 1 Introduction

Head and neck squamous cell carcinoma (HNSCC) accounts for the majority of head and neck cancers and represents the sixth most common cancer worldwide. It arises from the mucosal epithelium of the upper aerodigestive tract, including the oral cavity. Major risk factors include tobacco use, alcohol consumption, betel leaf chewing, and human papillomavirus (HPV), particularly in pharyngeal cancers. The use of areca nut or betel quid products is strongly associated with high rates of oral cavity cancer in India, where it is the most common malignancy among men. Although dysplasia may progress to invasive HNSCC, diagnosis is frequently made at an advanced stage. Treatment is typically multimodal, involving surgery followed by chemoradiotherapy (CRT). Cetuximab, an epidermal growth factor receptor (EGFR) monoclonal antibody, it may be used in combination with radiotherapy for HPV-negative HNSCC (1).

Patients diagnosed with advanced-stage tumours often demonstrate invasion of surrounding tissues, regional lymph node involvement, and distant metastasis, as well as an increased lifetime risk of second primary malignancies. Oral squamous cell carcinoma most commonly metastasises to the cervical lymph nodes (2). HNSCC spreads via direct extension, lymphatic dissemination, or haematogenous routes. Distant metastasis remains uncommon, occurring in approximately 4–26% of cases. The lungs, bone, and liver are the most frequent metastatic sites, while less common locations include the skin, bone marrow, brain, kidneys, eyes, and heart. The prognosis for patients presenting with distant metastases is generally poor. Bone metastases most frequently involve the spine, skull, ribs, and axial skeleton (3). For patients with metastatic disease receiving best systemic therapy, median overall survival is approximately 10 months. Duprez et al. reported that 4.1% of HNSCC patients developed bone metastases (4). Fluorodeoxyglucose positron emission tomography–computed tomography (FDG PET-CT) is the recommended imaging modality for detecting skeletal metastases (5).

The objective of this article is to report a rare case of femoral metastasis arising from HNSCC and to systematically review the existing literature on previously reported cases. We aim to analyse patient demographics, geographic distribution, risk factors, and clinical outcomes.

## 2 Case report

**Figure 1:**
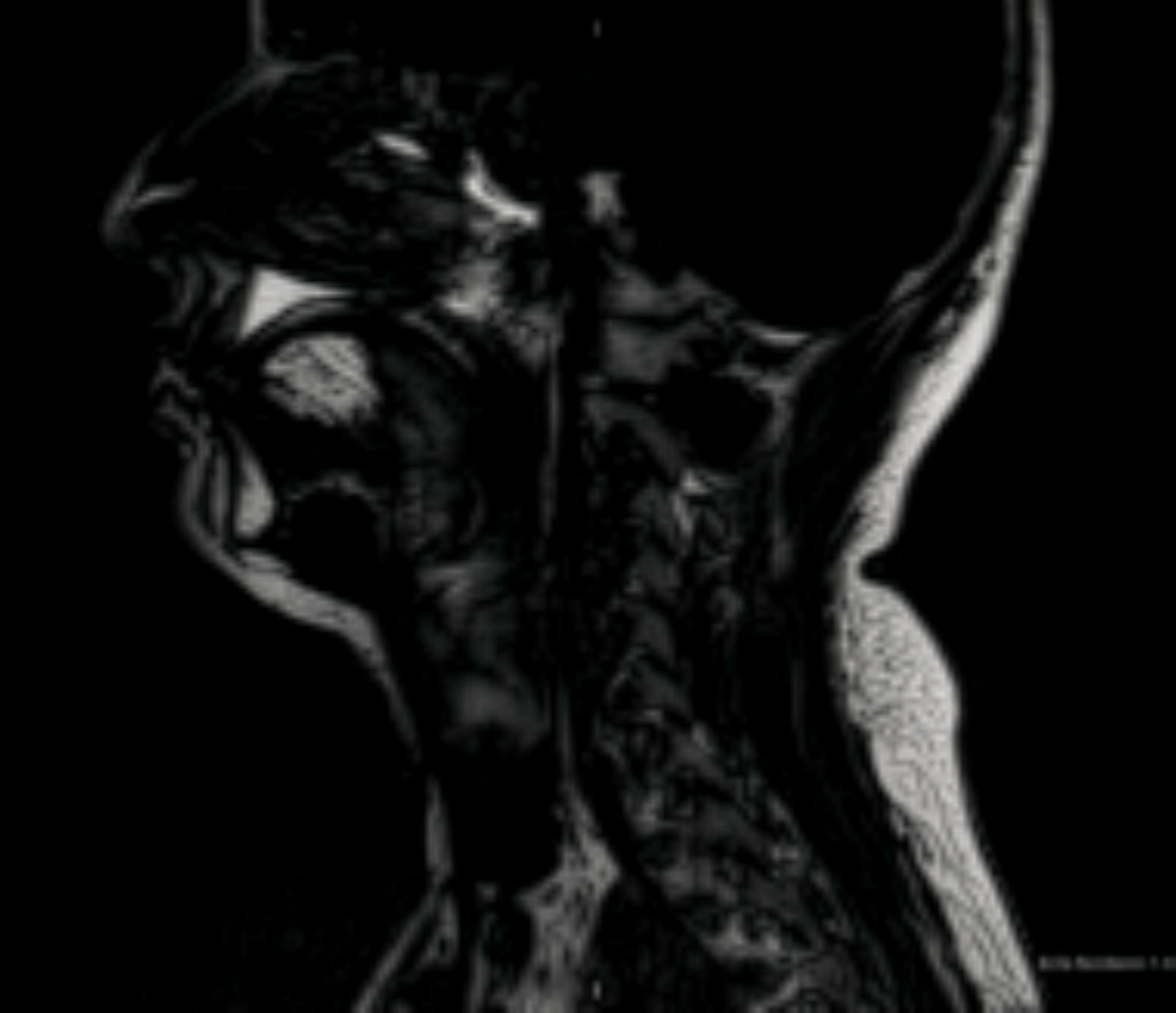
MRI Neck sagittal (arrow pointing to tongue base tumour).

**Figure 2:**
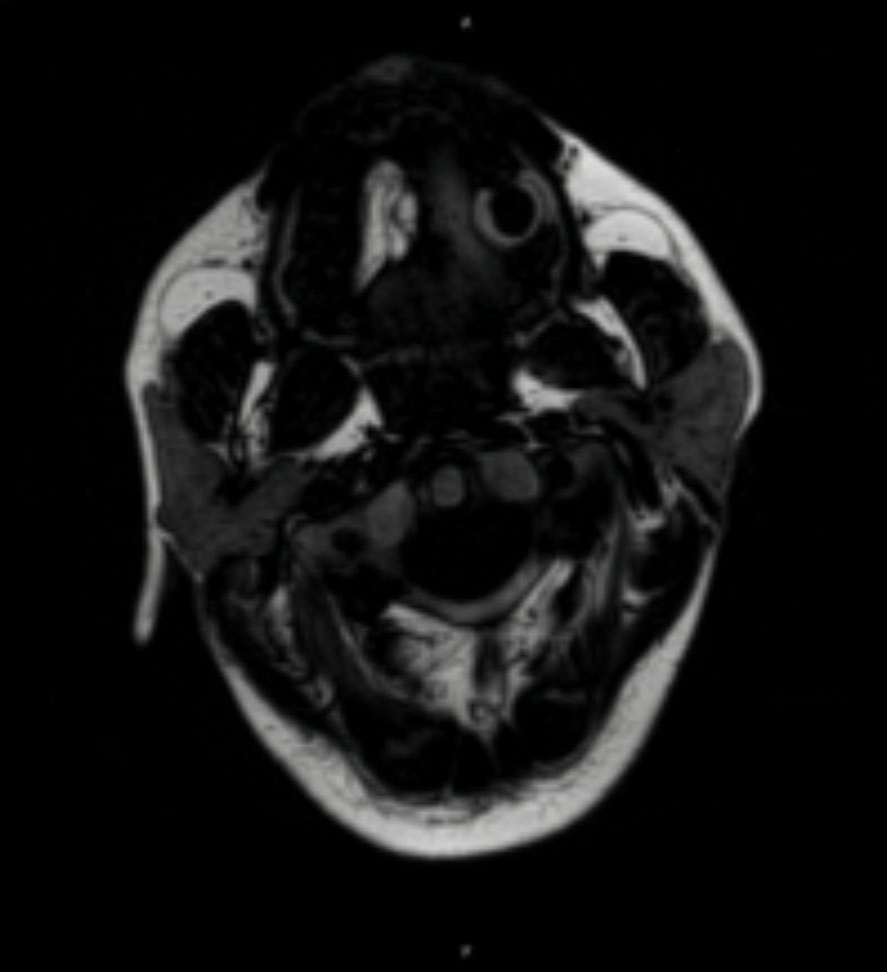
MRI Neck transverse (arrow pointing to tongue base tumour).

**Figure 3:**
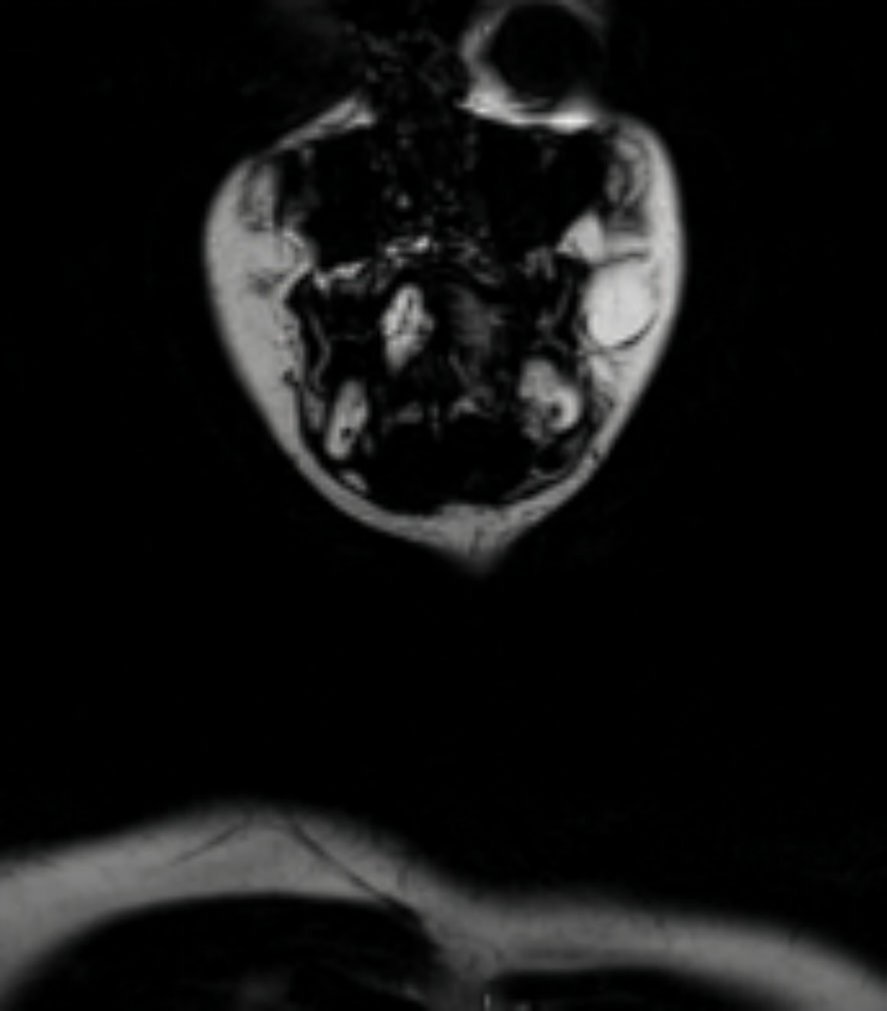
MRI Neck transverse (arrow pointing to tongue base tumour).

A 45-year-old Indian male with a history of smoking, moderate chronic obstructive pulmonary disease (COPD), and psoriasis presented with a six-month history of a painful ulcer on the right lateral aspect of the tongue. Biopsy confirmed poorly differentiated squamous cell carcinoma. Imaging demonstrated a 19 × 8.5 mm lesion without nodal or distant metastases, and the tumour was staged as T2N0. (American Joint Committee on Cancer (AJCC) 8^th^ edition staging). The patient underwent right partial glossectomy, right selective neck dissection, and reconstruction using a radial forearm free flap. Histopathological examination revealed a 17 mm, grade 3 squamous cell carcinoma with perineural invasion and clear surgical margins; all 22 cervical lymph nodes were negative. The patient elected for close surveillance rather than adjuvant therapy (**Figures 1**–**3**).

Six months later, the patient developed a contralateral left neck nodal recurrence. A left selective neck dissection demonstrated extensive nodal disease with extracapsular spread (11 of 42 nodes positive; pN3b). He subsequently received adjuvant chemoradiotherapy (66 Gy in 30 fractions) with cisplatin, later switched to carboplatin due to ototoxicity.

**Figure 4:**
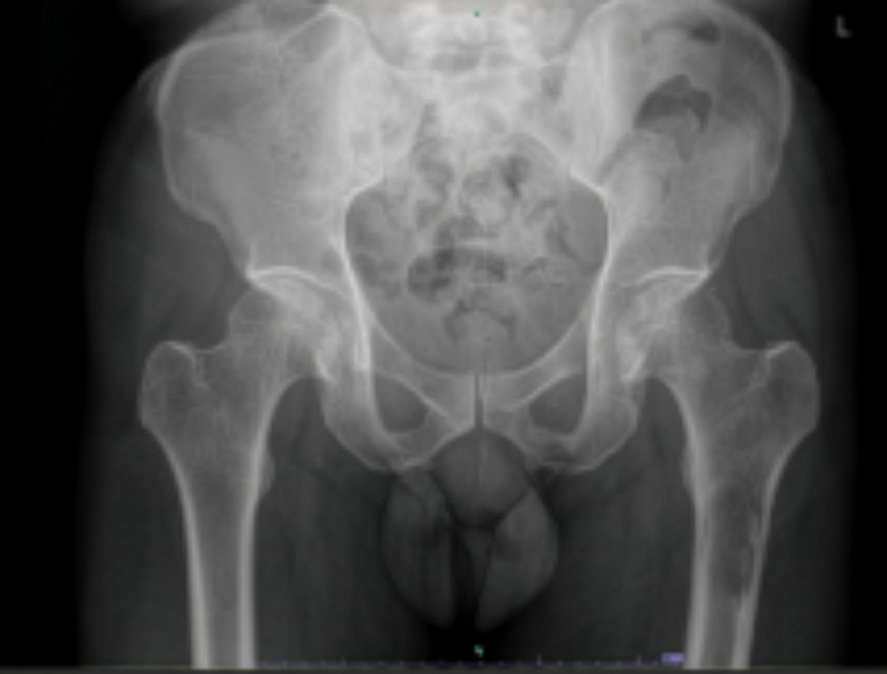
X ray Pelvis AP (arrow pointing to metastatic lesion in left femur).

**Figure 5:**
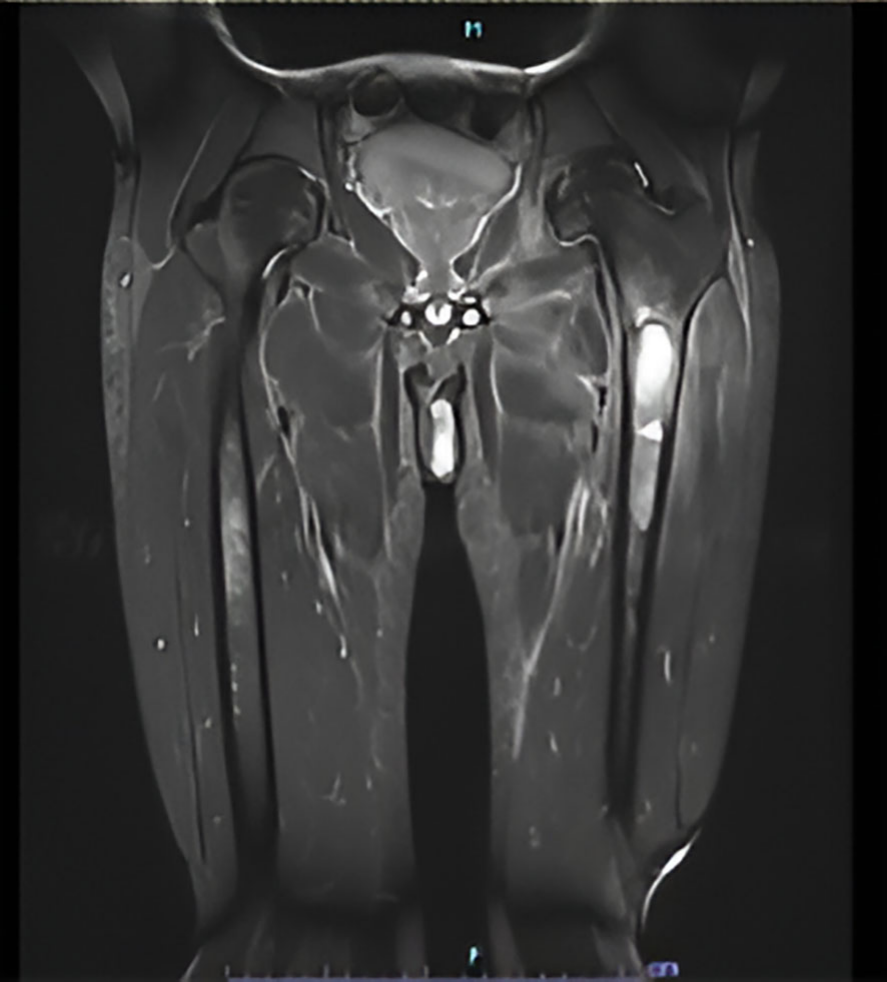
MRI Pelvis coronal (arrow pointing to metastatic lesion in left femur).

**Figure 6:**
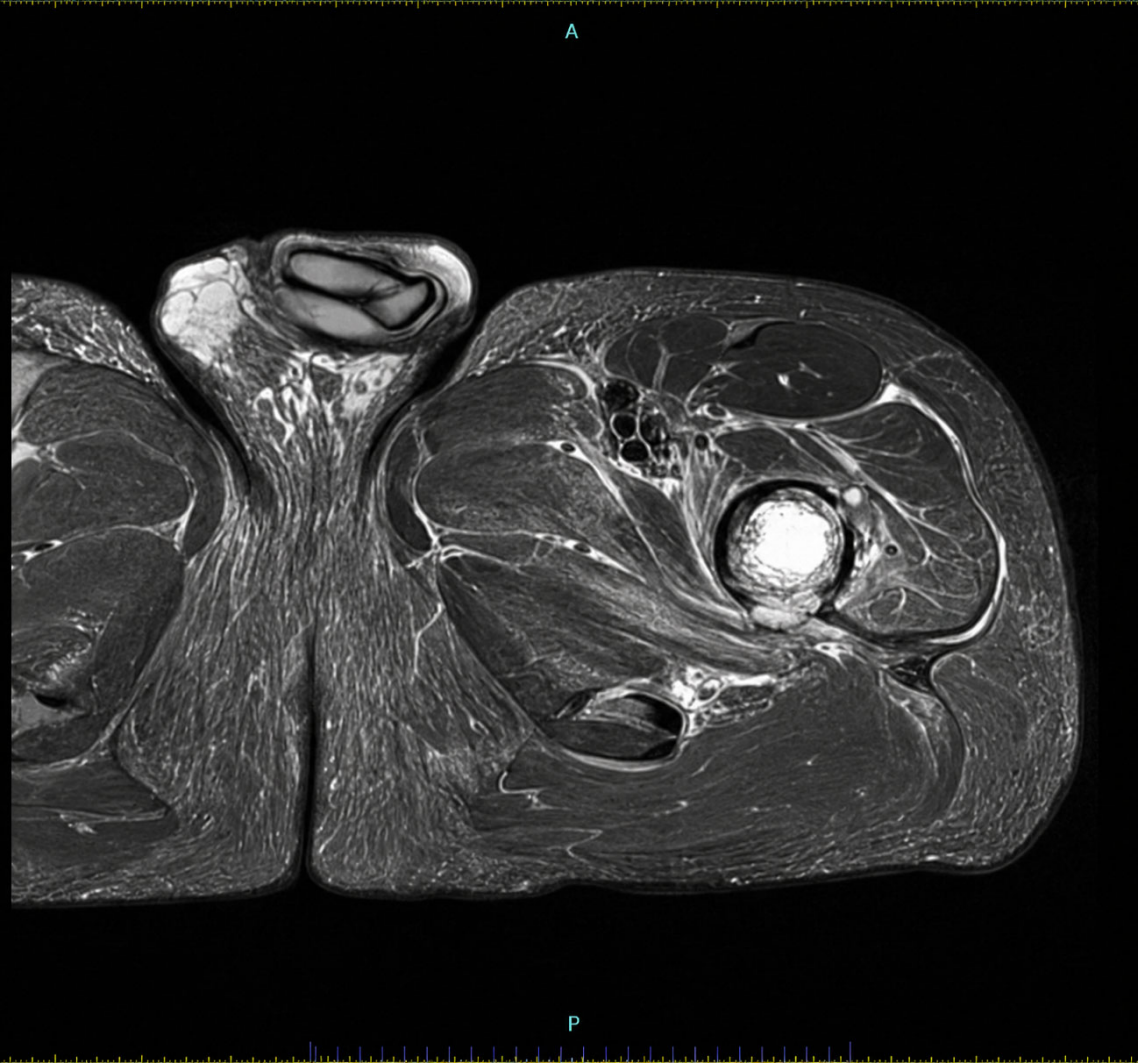
MRI Pelvis transverse (arrow pointing to metastatic lesion in left femur).

**Figure 7:**
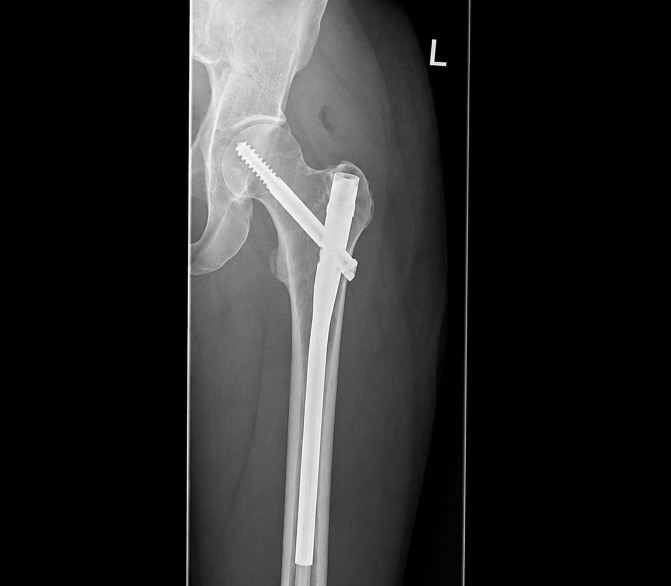
X ray Left femur AP showing intramedullary nail in left femur.

Despite treatment, surveillance imaging revealed progressive distant metastatic disease involving both lungs, followed by adrenal, rib, and vertebral metastases (**Figures 4**–**6**). Palliative systemic therapy with nivolumab achieved temporary disease stability before further progression. Subsequent imaging identified a metastatic lesion in the left femoral shaft with features of an impending pathological fracture. Prophylactic intramedullary fixation was performed, followed by palliative radiotherapy (**Figure 7**).

**Figure 8:**
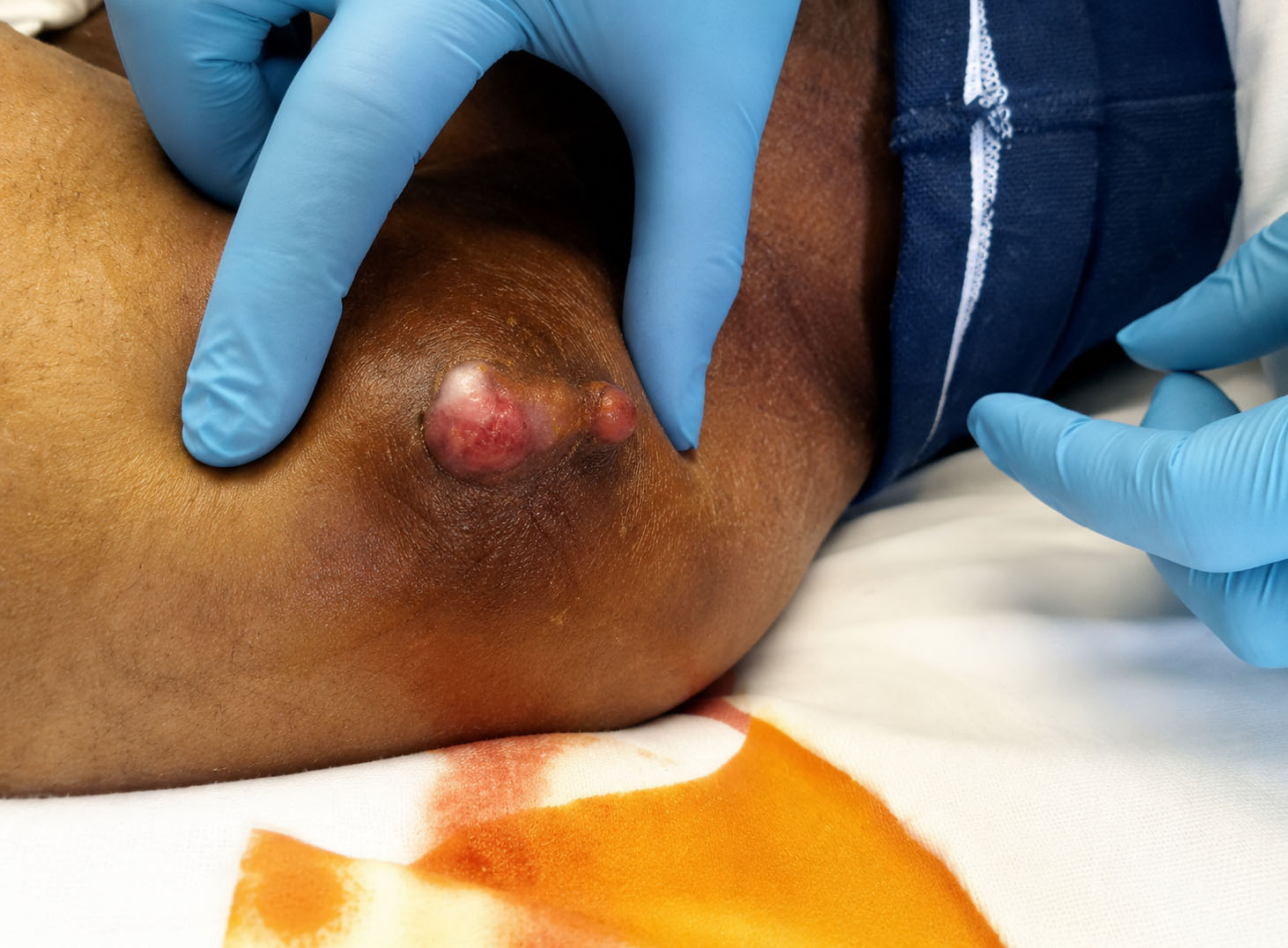
Picture of left hip showing suspected deep prosthetic infection.

Eight weeks later, the patient returned to theatre with suspected deep prosthetic infection (**Figure 8**). Intra-operative findings revealed extensive necrotic metastatic infiltration of the fascia lata and gluteus medius, with a sinus tract extending to the femoral metastatic lesion and complete loss of cortical bone. A superimposed infection was present. Histological analysis confirmed poorly differentiated squamous cell carcinoma with extensive infiltration of adipose tissue, muscle, and bone.

**Figure 9:**
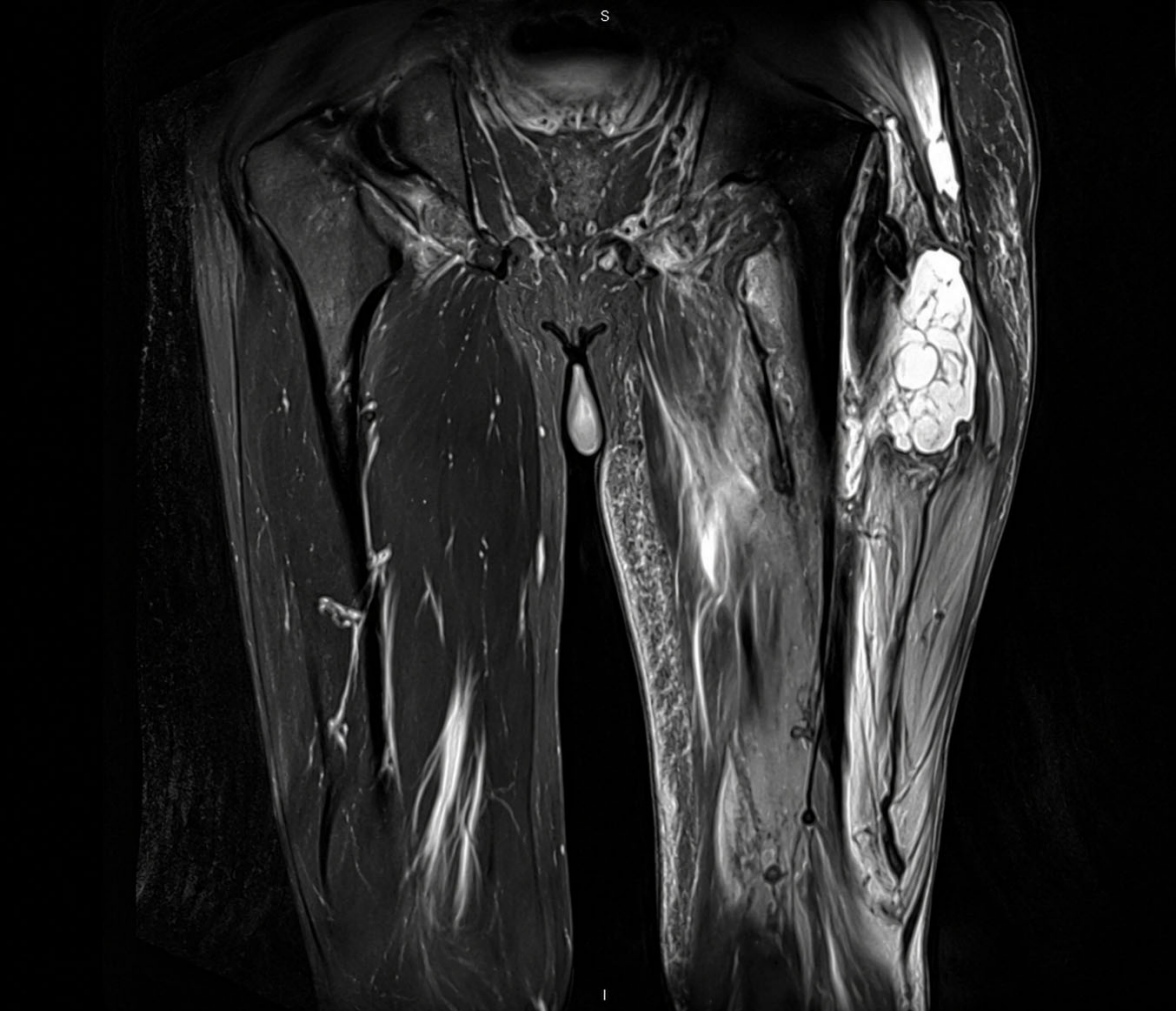
MRI Pelvis coronal arrow pointing to growth of left femur metastasis.

Post-operative magnetic resonance imaging demonstrated significant progression of the femoral metastasis (**Figure 9**). Given the rapid disease progression, limited prognosis, and poor functional reserve, further radical orthopaedic intervention was deemed inappropriate. Patient treatment was changed to best supportive care and discharged home with palliative services.

### 2.1 Summary of events in timeline format

- Patient presents with six-month history of a painful ulcer on the right lateral aspect of the tongue. He underwent right partial glossectomy, right selective neck dissection, and reconstruction using a radial forearm free flap.
- Six months from initial presentation, the patient developed a contralateral left neck nodal recurrence. A left selective neck dissection with adjuvant chemoradiotherapy.
- 12 months from initial presentation, surveillance imaging revealed progressive distant metastatic disease involving both lungs, followed by adrenal, rib, and vertebral metastases.
- 15 months from initial presentation imaging identified a metastatic lesion in left femoral shaft. Prophylactic surgery was performed shortly after identification.
- 17 months from initial presentation, patient returned to theatre with suspected deep prosthetic infection. Shortly after imaging confirmed progression of femoral metastasis and patient was transition to best supportive care.

This case illustrates the aggressive behaviour and rare skeletal metastatic pattern of tongue squamous cell carcinoma, highlighting the challenges of surveillance, treatment sequencing, and palliative decision-making in advanced disease.

### 2.2 Outcome/follow up

At the time of writing, the patient remains alive, five months following the diagnosis of femoral metastasis.

## 3 Methodology

### 3.1 Study design

This study comprises a case report and a systematic review of the literature. The systematic review was conducted in accordance with the Preferred Reporting Items for Systematic Reviews and Meta-Analyses (PRISMA) guidelines (Supplementary Material 1). This systematic review has been registered in PROSPERO (Registration number CRD420261293179).

### 3.2 Search strategy and study selection

An electronic literature search was performed across five bibliographic databases: PubMed, Scopus, ScienceDirect, the British Medical Journal, and Google Scholar (case reports). No date restrictions were applied due to the limited number of published cases. Two authors (H.C. and J.L.) independently screened articles and performed quality assessment of the included studies. We used the Joanna Briggs Institute (JBI) Checklist for Case Reports as our quality appraisal tool. Final search of literature was completed in January 2026.

### 3.3 Eligibility criteria

#### 3.3.1 Condition studied

Head and neck squamous cell carcinoma with femoral metastasis.

#### 3.3.2 Exposure

Histologically confirmed HNSCC with femoral metastasis.

#### 3.3.3 Comparator

Not applicable.

Inclusion criteria comprised of case reports written in English, describing patients with histologically confirmed HNSCC as the primary malignancy and documented femoral metastasis. No specific time frame for published case reports. Patients of any age, gender, ethnicity and co-morbidities.

Exclusion criteria included case reports not written in English, patients without histologically confirmed HNSCC as primary malignancy and no femoral metastasis.

## 4 Results

**Figure 10:**
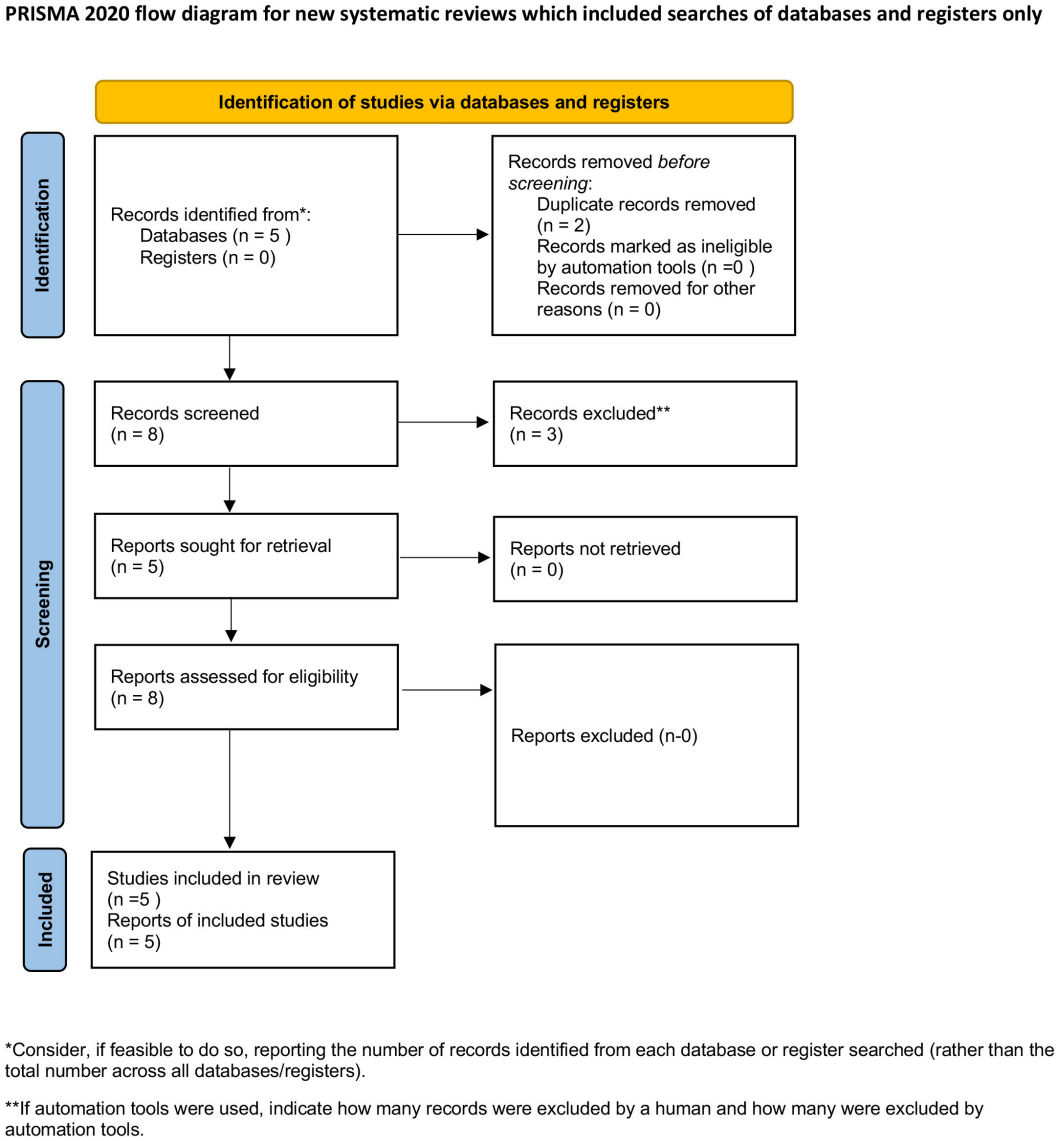
PRISMA flowchart outlining the study search.

A total of five published cases of femoral metastasis from HNSCC were identified in the literature (**Figure 10**). When combined with the current case, six cases were included in the final analysis. Please see **Table 1**.

**Table 1:** This table represents data from our systematic review (Case reports in which the following could not be found we have written NA).

Study	Year	Country	Age	Gender	Risk factors	Primary tumour site	Stage	Initial treatment	Time to first recurrence	Site of first recurrence	Metastasis site	Treatment after metastases
Chahal et al.	2026	UK	45	Male	Smoker	Tongue SCC	T2N0M0	Surgery	6 months	Contralateral submandibular nodes	Pulmonary, ribs, mesentery, femur	Surgery, chemoradiotherapy
Dudde et al.	2023	Germany	60	Female	NA	Mouth and tongue SCC	NA	Surgery adjuvant chemo radiotherapy	19 years	Floor of mouth	Mandible, femur, bones	Palliative radiotherapy
Hull et al.	2021	USA	57	Male	Smoker	Neck SCC	T2N2bM0	Palliative chemoradiotherapy	NA	NA	NA	Palliative chemoradiotherapy
Agrawal et al	2019	India	40	Male	NA	Tongue SCC	NA	Surgery adjuvant radiotherapy	1 year	Femur	Femur	NA
Anila et al	2018	India	62	Male	Smoker and Betel leaf use	Gum SCC	T2N2M0	NA	8 months	Femur	Femur	Palliative radiotherapy
Hampton et al	2013	UK	79	Female	NA	Tongue and neck SCC	T2N2M0	Chemoradiotherapy	11 months	Femur	Femur	Palliative radiotherapy

*Records identified from, Pubmed, Scopus, Science Direct, British Medical Journal and Google scholar case reports. Excluding our case report.

**Reasons reports excluded.

Reason 1- Metastasis not in femur (n =2).

Reason 2 – HNSCC not primary source of malignancy (n = 1).

## 5 Discussion

Among the six cases identified between 2013 and 2026, four originated from India, one from the United Kingdom, one from Germany, and one from the USA (6–10). Two-thirds of patients were of Indian ethnicity, reflecting the higher regional prevalence of HNSCC. Smoking was reported as a risk factor in 50% of cases, while the remaining reports did not document recognised risk factors, suggesting either incomplete reporting or no risk factors identified.

Patient age ranged from 40 to 79 years, with a mean age of 57 years and a median age of 58.5 years. This is younger than the traditionally reported age group for HNSCC, which typically affects individuals aged 60 years and older. However, recent epidemiological trends indicate increasing incidence among younger patients (11). Most patients were male (4/6, 66.7%), consistent with known epidemiological patterns.

The tongue was the most common primary tumour site, accounting for four cases (66.7%), followed by the floor of mouth and neck. Of the cases with available staging information, four patients (66.7%) had T2 disease with nodal involvement, reinforcing the established association between nodal metastasis and haematogenous dissemination. Additional metastatic sites included the mandible, ribs, lungs, and mesentery.

The time to recurrence or metastasis varied widely, ranging from six months to 19 years. Early recurrence (≤12 months) occurred in 66.7% of patients, suggesting aggressive tumour biology, although one case demonstrated extremely late metastatic presentation.

Initial treatment strategies varied, with surgery performed in four cases, either alone or combined with adjuvant radiotherapy or chemoradiotherapy. Following the diagnosis of distant metastasis, treatment intent was predominantly palliative. Palliative radiotherapy or chemoradiotherapy was administered in 83.3% of cases, reflecting limited curative options once distant disease is established.

Most case reports lacked detailed follow-up protocols, radiotherapy dosing, chemotherapy regimens, or survival outcomes following femoral metastasis diagnosis. The case reports also neglect to mention which edition of AJCC staging is used. National multidisciplinary guidelines recommend follow-up at least every two months for the first two years and every three to six months thereafter, for a minimum of five years. Clinical assessment should include thorough examination and endoscopic evaluation, with MRI and PET-CT imaging used when recurrence is suspected (12). While orthopaedic guidelines exist for the management of femoral metastases, there are currently no HNSCC-specific recommendations. Current British Orthopaedic Association (BOA) guidelines for management of metastatic bone disease suggests surgical interventions should outlast the lifetime of the patient.

As mentioned previously HNSCC typically spreads via direct extension, lymphatic dissemination, or haematogenous routes. Potentially the mechanism for femoral metastasis may be via haematogenous spread but this has not yet been further investigated.

## 6 Limitations and strengths

To our knowledge, this is the first systematic review summarising published case reports and case series describing femoral metastases from HNSCC. This study provides a comprehensive synthesis of available clinical data and explores potential demographic patterns and prognostic implications.

Limitations include the small sample size, reliance on case reports, and potential publication bias. Additionally, heterogeneity in staging systems, treatment approaches, and follow-up durations limits direct comparison across cases. Underreporting of femoral metastases in HNSCC is also likely. Most case reports did not state the overall survival rate after being diagnosed with femoral metastasis which would be useful to determine prognosis for future patients.

## 7 Conclusion

This systematic review summarises six reported cases of femoral metastasis from head and neck squamous cell carcinoma, emphasising the rarity, heterogeneity, and poor prognosis associated with this condition. Although HNSCC predominantly spreads locoregionally, distant metastasis, particularly to the femur, represents an advanced disease state with limited treatment options. Early recognition and timely palliative intervention are essential to optimise quality of life. Future multicentre studies and pooled analyses are required to better characterise risk factors, metastatic pathways, and optimal management strategies.

## Data Availability

The raw data supporting the conclusions of this article will be made available by the authors, without undue reservation.
